# Simulation study on radiation exposure of emergency medical responders from radioactively contaminated patients

**DOI:** 10.1038/s41598-021-85635-2

**Published:** 2021-03-17

**Authors:** Takakiyo Tsujiguchi, Yoko Suzuki, Mizuki Sakamoto, Kazuki Narumi, Katsuhiro Ito, Hiroshi Yasuda, Shinji Tokonami, Ikuo Kashiwakura

**Affiliations:** 1 Graduate School of Health Sciences, Hirosaki University, 66-1 Hon-cho, Hirosaki, 036-8564 Japan; 2 Advance Emergency and Critical Care Center, Hirosaki University Hospital, 5 Zaifu-cho, Hirosaki, 036-8562 Japan; 3 Research Institute for Radiation Biology and Medicine, Hiroshima University, 1 Kasumi 2-3, Minami-ku, Hiroshima, 734-8553 Japan; 4 Institute of Radiation Emergency Medicine, Hirosaki University, 66-1 Hon-cho, Hirosaki, 036-8564 Japan

**Keywords:** Occupational health, Risk factors

## Abstract

Emergency medical responders (EMRs) who treat victims during a radiation emergency are at risk of radiation exposure. In this study, the exposure dose to EMRs treating hypothetically contaminated patients was estimated using a Monte Carlo simulation, and the findings may be useful for educating EMRs and reducing their anxiety. The Monte Carlo simulation estimated radiation doses for adult computational phantoms based on radioactive contamination conditions and radiation dosages from previous studies. At contamination conditions below the typical upper limit of general Geiger–Müller survey meters, the radiation doses to EMRs were estimated to be less than 1 μSv per hour. In cases with greater contamination due to mishandling of an intense radioactive source (hundreds of GBq), the radiation doses to EMRs could reach approximately 100 mSv per hour. These results imply that a radiological accident with a highly radioactive source could expose EMR to significant radiation that exceeds their dose limit. Thus, authorities and other parties should ensure that EMRs receive appropriate education and training regarding measures that can be taken to protect themselves from the possibility of excessive radiation exposure. The results of this study may provide EMRs with information to take appropriate protective measures, although it is also important that they not hesitate to perform lifesaving measures because of concerns regarding radiation.

## Introduction

Radiation is widely used in medical and industrial fields. Thus, when nuclear or radiological accidents occur, facility workers and neighboring residents may be exposed to radioactive substances. One example is the Fukushima Daiichi Nuclear Power Plant (FDNPP) accident, which is a well-known nuclear disaster that was assigned the highest rating (level 7) on the International Nuclear and Radiological Event Scale (INES)^1–3^. During the FDNPP accident, > 170,000 nearby residents were forced to evacuate and emergency medical responders (EMRs) and health physicists were required to perform difficult tasks^2^. In addition to nuclear emergencies, there have been situations that involved contamination and exposure of large numbers of people because of the theft a radiation source, such as the Goiânia accident in Brazil and the Juarez accident in Mexico^4,5^. Improving the medical response to these radiation emergencies requires establishment of international standards for training EMRs and providing them with appropriate radiological protection equipment^6,7^. The exposure doses and patient contamination levels can be estimated using various analyses, which can guide the development of risk prediction models and related risk reduction strategies^8,9^. However, there is little discussion regarding the radiation dose that EMRs may receive from patients while managing a radiation emergency. One example is that contamination inspections are required to comply with the International Atomic Energy Agency (IAEA) technical documents^10^. It is also possible that a highly radioactive source may be powdered and may adhere to patients, as observed during the Goiânia accident. Thus, EMRs are inevitably exposed to radioactive substances from workers and evacuees who are contaminated during a nuclear disaster. In addition, doctors and nurses who provide emergency medicine in this setting have a particularly high risk of radiation exposure.

A recent report described survey responses from EMRs who were dispatched during the FDNPP accident and indicated that they were afraid of being exposed to radiation when they provided emergency care^11^. This anxiety during radiation emergencies may impair decision-making, despite the development of emergency response and personnel strategies. Therefore, if a standard exposure dose could be defined for EMRs, specialized education might be implemented to help manage their safety and anxiety, which would allow them to respond calmly and rationally to situations that may involve radiation exposure. This study involved simulation of exposure doses to EMRs who provide emergency care to hypothetical patients who were contaminated by radioactive substances, based on reported information from previous radiation emergencies. The exposure doses to EMRs were estimated using the Particle and the Heavy Ion Transport code System (PHITS)^12–14^, which can calculate photon transport based on various assumptions regarding the radiation emergency and contaminated patient.

## Methods

### Monte Carlo code used to simulate the effective dose

A PHITS-based Monte Carlo simulation was performed to estimate the radiation dose that EMRs could receive from contaminated patients^12–14^. The PHITS system can simulate radiation behavior and has been used for various research projects in the fields of medical physics and space engineering^15–17^. A benchmark study regarding photon simulation has been reported for energy ranging from 1 keV to 10 GeV^14^. This PHITS code was used for all physical simulations in the current study.

### Creating simulation geometry for the contaminated patient and EMR

**Figure 1:**
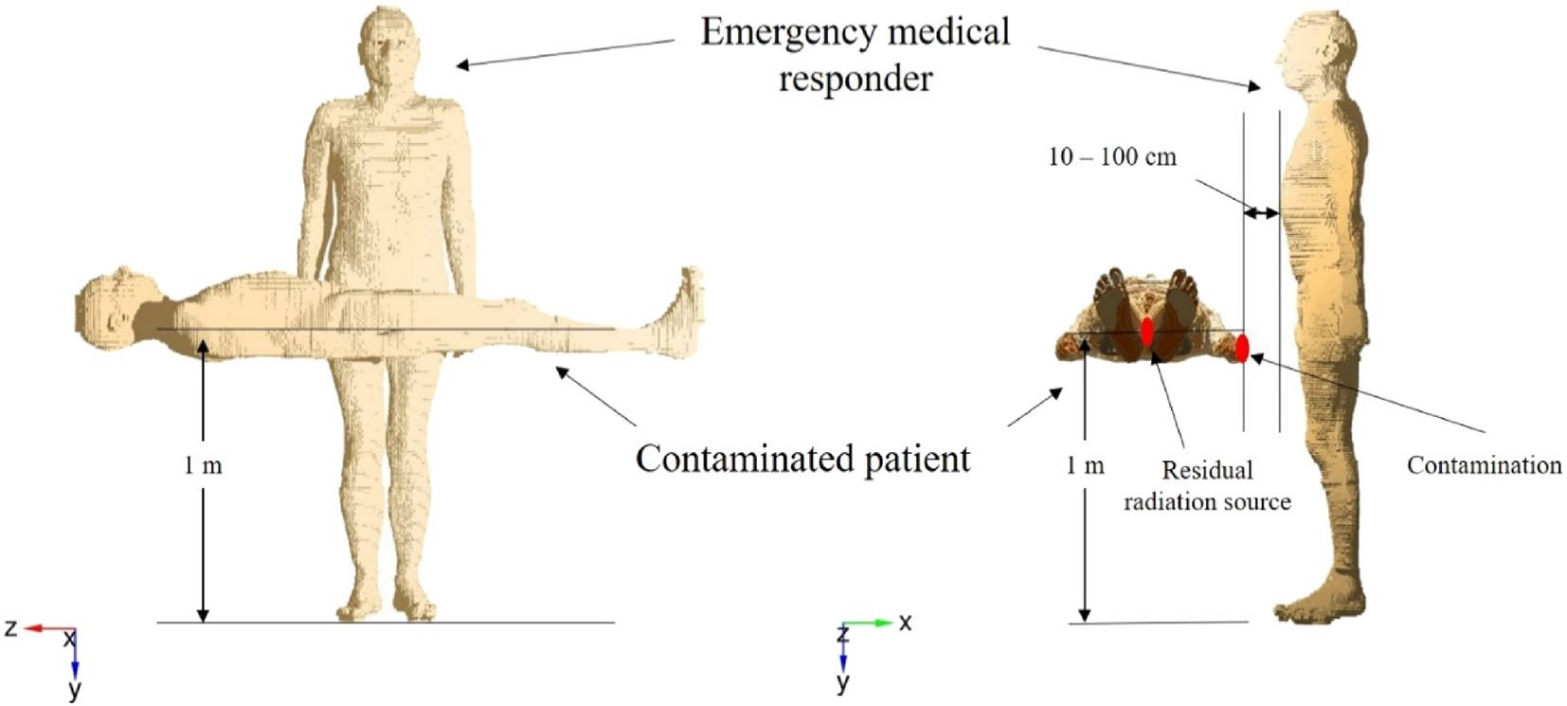
Geometry created using the particle and the heavy ion transport code system. The radionuclides can be set to any location, and the amount of contamination and the distance between the patient and the emergency medical responder can be changed arbitrarily.

An adult computational phantom was created in a virtual space for a hypothetically contaminated patient and an EMR. The adult computational phantom (Reference Computational Phantom–Adult Male) was from ICRP Publication 110^18^. Figure 1 shows the actual geometry images created using the PHITS code. Assuming that the contaminated patient was on a stretcher, the trunk center was placed 1 m above the ground and at a right angle to the EMR. Scenarios were established with adhesion of various radioactive substances to the patient’s upper arm or the presence of a radiation source within the patient’s abdominal cavity. We calculated the photon scattering from radioactive contamination and the absorbed dose for each of the EMR’s organs. Finally, the effective dose was estimated by integrating the absorbed doses for all organs and tissues. The number of simulation trials was set to 10^7^ per condition to ensure reliability of the Monte Carlo calculation, and the relative error for each trial was set to < 0.05 based on a previous report^19^. The simulation assumed that there was no scattering from the floor and walls.

### Creating the patient contamination and EMR exposure scenarios

Simulations of emergency medicine and radiation physics require careful consideration of the assumptions. The present study used two general scenarios that were based on previous radiation emergencies: (1) exposure from environmentally released radionuclides and (2) exposure from directly handling a radioactive source. The effective doses to the EMR were calculated for each scenario and the related assumptions are summarized in Table 1.

**Table 1:** Assumptions regarding radionucleotides that are attached to a contaminated patient in this study.

Scenario	Situations where responders are expected to be exposed	Radionuclide	Assumptions regarding contamination density	Shape of contamination	Past cases
1	**Exposure from environmentally released radionuclides** ・Radionuclides released from nuclear power plants ・External contamination of evacuees	^131^I	**Assuming 400 Bq/cm**^**2**^** contamination on the body surface** (approximately 100,000 cpm using a GM survey meter with a window area of 20 cm^2^, which is used to inspect evacuees for contamination)	A circular contamination area with a radius of 5 cm on the back of the hand	Chernobyl nuclear power plant accident (USSR, 1986) Fukushima Daiichi nuclear power plant accident (Japan, 2011)
		^134^Cs			
		^137^Cs			
2	**Exposure from directly handling a radioactive source** ・Illegal handling of a source without proper protection ・Misuse of a source in industry/medicine	^60^Co	Assuming a 10-GBq source on the body surface	Point-source-like contamination on the back of the hand with a radius of 1.25 cm	Goiânia accident (Brazil, 1987)
		^137^Cs			Juarez accident (Mexico, 1983)
		^241^Am	Assuming a 185-GBq contamination	A circular contamination area with a radius of 5 cm on the back of the hand	Hanford accident (USA, 1976)
		^192^Ir	Assuming a 137-GBq source in the body	Assuming that a φ1 mm × 5 mm radiation source remains in the abdomen	Indiana accident (USA, 1992)

During nuclear disasters, such as the Chernobyl nuclear power plant (ChNPP) accident and the FDNPP accident, environmentally released radioactive substances (e.g., iodine and cesium) contaminate members of the general population. We defined this situation as Scenario 1 and considered how health physicists might be exposed when they inspected contaminated residents. The assumed contamination level was based on decontamination following Operational Interventional Level 4 (OIL4) from the IAEA technical document^10^. Scenario 1 in Table 1 shows the assumptions regarding contamination concentration and shape.

Scenario 2 involved body surface contamination with radioactive materials that was caused by the theft or mishandling of medical or industrial sources, which occurred during the Goiânia, Juarez, Hanford, and Indiana accidents. Assumptions regarding the amount of contamination in Scenario 2 were based on previously reported information^20,21^ and the radioactivity of radiation sources from common medical or industrial applications. This scenario is not a faithful reproduction of cases from previous radiological accidents, and detailed information regarding the size and area of contamination is shown in Table 1.

### Verification of simulation accuracy

Sato et al.^13^ have reported verified benchmarks for radiation and energy that can be used for PHITS-based simulations. Given the importance of confirming deviation from the actual measurement during a physical simulation study, our research group prepared phantoms and other conditions in the reproducible range in an attempt to verify accuracy. We placed a human phantom (PBU-60; Kyoto Giken Kogyo Co., Japan) on a stretcher and attached a sealed ^60^Co radiation source (57,200 Bq at the measurement date) to the phantom’s upper arm. The absorbed dose in the air was then measured using a 3-in × 3-in NaI(Tl) scintillation spectrometer (EMF-211; EMF Japan Co., Japan) at points equidistant from the contamination site, with measurements performed at 10-cm intervals (10–100 cm) from the contaminated area. The absorbed dose in the air was measured three times over 5-min periods. Similarly, at a distance of 10 cm from the contaminated site, the spectrometer was installed to perform five readings at 40 cm, 70 cm, 100 cm, 130 cm, and 160 cm from the floor. The same geometry and cobalt radiation source were also used for the PHITS-based simulations. Accuracy was verified by comparing the measured values to those from the simulation model.

## Results

### Verifying the accuracy of the simulation model

**Figure 2:**
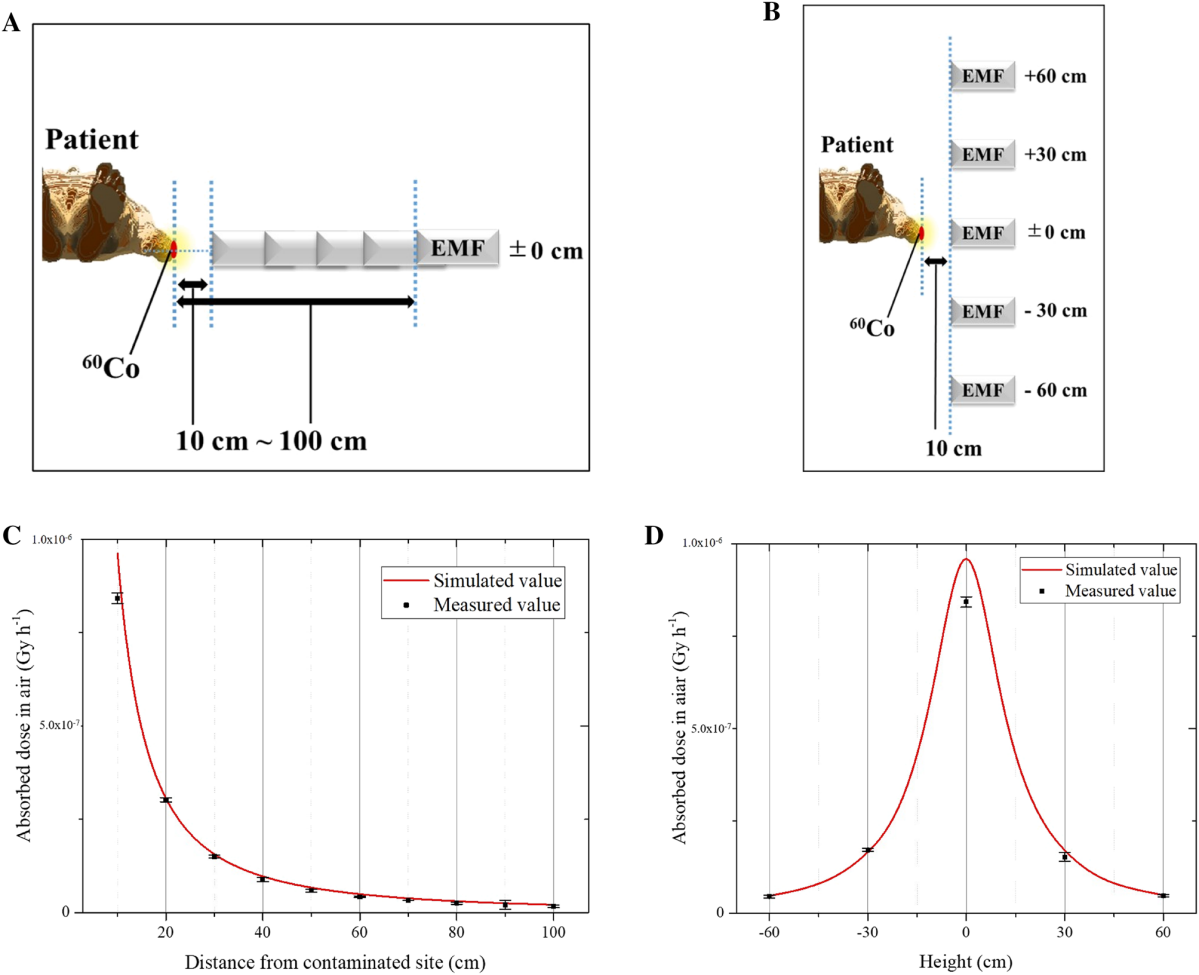
Verifying the accuracy of the simulation model. (**A**) The outline when the spectrometer was set at 10-cm intervals (10–100 cm) from the contaminated site. (**B**) The outline when the height of the spectrometer was changed to a distance of 10 cm from the contaminated site. (**C**) Comparing the measured and simulated values when the spectrometer was set at 10-cm intervals (10–100 cm) from the contaminated site and (**D**) when the height of the spectrometer was changed to a distance of 10 cm from the contaminated site.

The measurement and simulation results are shown in Fig. 2. Comparisons of the measured and simulated spectrometer values (10-cm intervals, 10–100 cm from the contaminated site) revealed a relative error rate of < 14% at all points (Fig. 2A). Similarly, the relative error rate of the simulated value was < 14% when compared to the spectrometer results at various height and a distance of 10 cm from the contaminated site (Fig. 2B). At each point, the simulated values of the absorbed dose in the air did not underestimate the measured values. Thus, based on acceptable accuracy findings, the simulations were performed to collect the following results.

### Estimated exposure dose to EMRs treating contaminated patients

**Figure 3:**
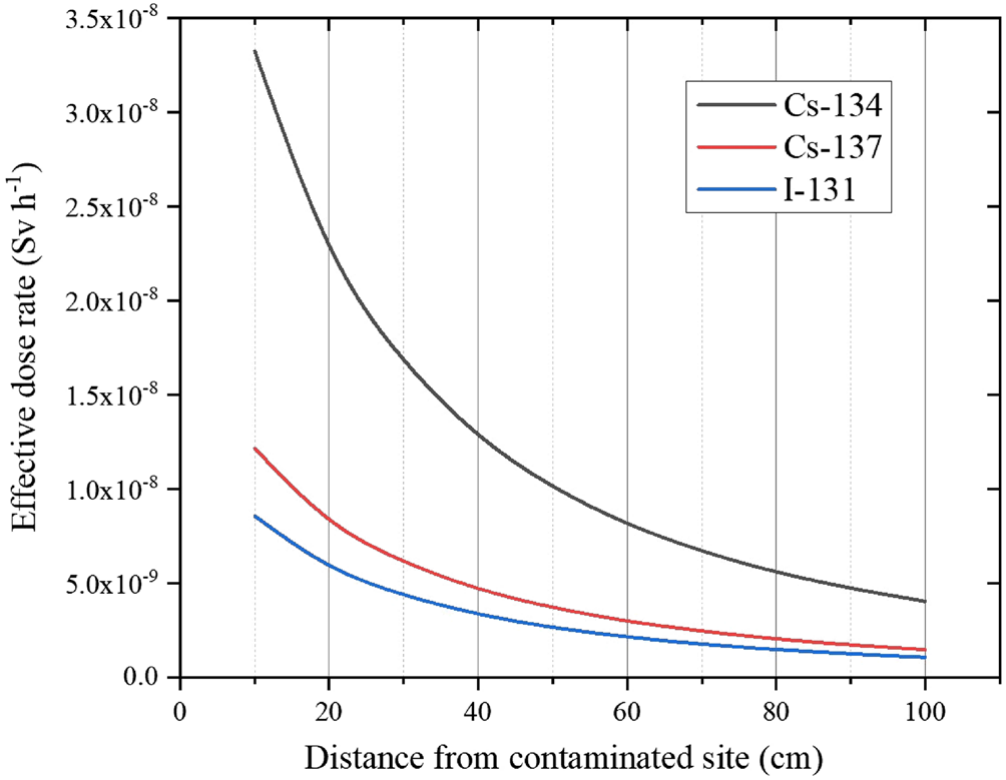
Exposure doses of an emergency medical responder who cares for a patient who was contaminated with radionuclides that are expected to be released during a nuclear disaster. Each of the three sources is on the surface of the body and has an intensity of 400 Bq cm^−2^. Table 1 shows the detailed assumptions for this Scenario (Scenario 1).

**Figure 4:**
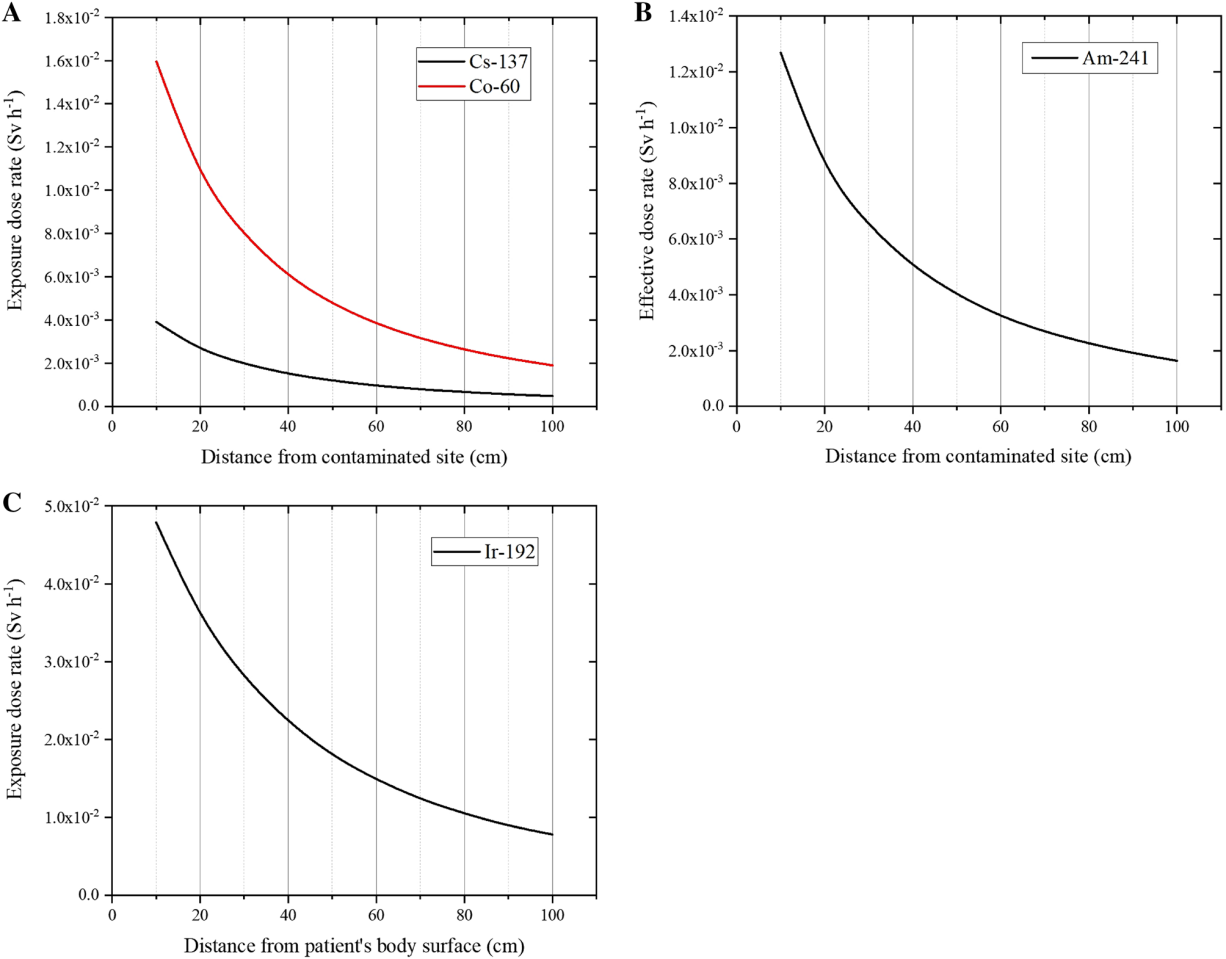
Exposure doses of an emergency medical responder who cares for a patient with highly radioactive contamination during a radiological accident. Table 1 shows the detailed assumptions for these scenarios (Scenario 2). (**A**) Radiological accidents associated with the theft of medical/industrial radiation sources, assuming a 10-GBq source. (**B**) Radiological accidents caused by mishandling of industrial radiation source, assuming a 185-GBq source. (**C**) Radiological accidents related to mishandling of a medical radiation source (e.g., for brachytherapy), assuming a 137-GBq source.

Figures 3 and 4 show the simulation results. In situations where the patient’s contamination was below the upper limit of a general Geiger–Müller survey meter, the maximum external exposure dose to the EMR was approximately 33 nSv per hour at a distance of 10 cm from the contamination (Fig. 3). In situations where the patient might have been contaminated with a powder from a radiation source, the maximum external exposure dose to the EMR was likely less than 16 mSv per hour (Fig. 4A). In situations involving a patient contaminated by high-capacity radiation sources, such as therapeutic radiation sources, the maximum external exposure dose to the EMR was approximately 48 mSv per hour (Fig. 4B). Assuming contamination with ^241^Am based on the Hanford accident (several hundred GBq), the maximum external exposure dose to the EMR was approximately 12 mSv per hour (Fig. 4C).

### Estimated times to reach various dose limits

**Figure 5:**
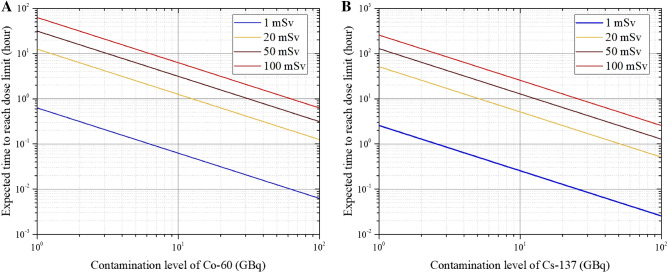
Exposure doses of an emergency medical responder caring for a patient contaminated by a highly radioactive radiation source at a distance of 10 cm for each contamination amount. When the radionuclide is (**A**) ^60^Co or (**B**) ^137^Cs, a point-source-like contamination (radius 1.25 cm) is assumed on the back of the hand (see Scenario 2 in Table 1).

Based on our simulation results, Table 2 summarizes the estimated times to reach various dose limits during planned or emergency exposures that are presented in ICRP Publication 103^22^. Fig. 5 shows the exposure dose of an EMR at a distance of 10 cm from the contaminated site when treating a patient in Scenario 2 (theft or mishandling of a medical or industrial radiation source). The IAEA recommends that the average planned occupational exposure dose limit over 5 years should be ≤ 20 mSv per year, and that it should not exceed 50 mSv in any year. Under emergency exposure situations, reference exposure dose limits of 100 mSv, 500 mSv, and 1000 mSv were selected. Based on the simulation results from the two scenarios (Table 1), the expected times to reach exposure dose limits of 1 mSv, 20 mSv, 50 mSv, or 100 mSv were calculated when treating the patient at a distance of 10 cm from the contaminated site.

**Table 2:** Expected time to reach various dose limits.

	Radionuclides	Expected time to reach dose limits			
		1 mSv ( min)	20 mSv	50 mSv (h)	100 mSv (h)
Scenario 2 (from Table 1)	^60^Co	3.76	1.24 h	3.13	6.27
	^137^Cs	15.25	5.11 h	12.79	25.58
	^241^Am	4.73	1.57 h	3.94	7.88
	^192^Ir	1.25	25.06 min	1.04	2.09

The expected times were derived from the dose rates at a distance of 10 cm from the patient.

We found that, when the patient’s contamination exceeded OIL4, which is expected during a nuclear disaster, the expected time for the EMR to reach an exposure dose of 1 mSv was > 1000 h. However, when the patient was contaminated in Scenario 2 (theft or mishandling of a radiation source), the expected time for the EMR to reach an exposure dose of 1 mSv ranged from a few minutes to tens of minutes. In a scenario involving ^192^Ir, an ERM who treated a contaminated patient at a distance of 10 cm for approximately 2 h would reach an exposure dose of approximately 100 mSv.

## Discussion

Based on the “as low as reasonably achievable” (ALARA) principle, the exposure dose to the public and professionals should be reduced as much as possible under the specific circumstances^22–24^. The ALARA principle is also applicable during radiation emergencies, although its applicability is complicated by the priority of saving the patient’s life in these emergencies, and it is important to develop appropriate treatment strategies that incorporate exposure dose evaluations^25,26^. Therefore, although it is not difficult to strictly implement the ALARA principle, this must also factor in the high value of the human life that is at stake in emergency situations. For example, doctors, nurses, and health physicists can conceivably be called upon to treat contaminated victims and evacuees in an emergency situation, which makes it difficult to ensure sufficient distance and time limitations in situations with unknown contamination. Wearing protective clothing can make it difficult to read a personal dosimeter, and even if the dosimeter has an alarm function, the alarm merely indicates that the set dose has been reached. Therefore, simply wearing a personal dosimeter does not predict dose exposure during a radiation emergency, and additional strategies and planning are needed to guide appropriate measures. For example, our findings might be useful for developing guidelines regarding appropriate personnel replacements and limits to activities at the disaster location^8^.

The results of this simulation study were obtained using the PHITS code and established benchmark values. Relative to actual measured values, the simulation values were comparable and did not underestimate the actual values, with a sufficiently reliable relative error rate (< 14%) (Fig. 2). Based on the simulation results, an EMR who treats a patient with contamination exceeding OIL4 would not realistically exceed the occupational exposure limit, regardless of the time spent treating the patient (Fig. 3, Table 2). However, in cases involving theft or mishandling of radiation sources for medical and industrial applications, the EMR might receive exposure doses of 1–100 mSv (Fig. 4, Table 2). The exposure dose of an EMR who provides radiation emergency medicine is the exposure dose when the contaminated area of the victim is not decontaminated at all. It is necessary to keep in mind that the exposure dose of both the patient and EMR can be reduced by performing appropriate decontamination after the patient's lifesaving measures are taken. Accidents in which a high-radioactivity source is attached to a patient are rare. It is unlikely that exposure to EMR by radioactive substances attached to the patient will cause a deterministic effect. Still, it is important to control the exposure dose for radiological protection.

Several studies have identified anxiety regarding radiation among medical professionals and the importance of education regarding radiological protection. For example, Sato et al. conducted a questionnaire survey of nurses working at core hospitals in Fukushima Prefecture after the FDNPP accident and reported that many nurses were considering retirement or migration because of anxiety regarding radiation and related health effects^27^. Akashi et al. have also recommended promptly providing radiological protection information to Disaster Medical Assistance Teams^28^. Therefore, it is important to provide radiological protection and education to EMRs who respond to radiation emergencies. The 2020 IAEA guidelines also highlight the importance of obtaining assistance from medical physicists with experience in radiation emergencies^29^. Thus, improving the knowledge, anxiety, and capabilities of EMRs is critically important to their radiological protection.

The results shown in Figs. 4 and 5 may be useful reference data for estimating EMRs' exposure doses based on different scenarios involving medical and industrial radiation sources. Although these events are rare, they are not impossible, as observed during the Goiânia accident (in Brazil) and the Indiana accident (in the US). There have also only been two level 7 accidents based on the INES scale, which were the ChNPP accident and the FDNPP accident. Nevertheless, over a 30-year period, there has been > 600 radiological accidents with > 2,000 incidents that involved exposed/contaminated victims^30^. The possibility of other incidents, such as nuclear terrorism, also further highlights the importance of providing appropriate radiological protection for EMRs.

Although radiological accidents or nuclear disasters are rare, they have enormous effects on medical professionals and the general population. Therefore, radiological protection strategies and education forEMRs and related institutions should be considered before these events occur. We believe that the findings of this study will be useful for guiding the development of these radiological protection strategies and related education.
